# Drug resistant cells with very large proliferative potential grow exponentially in metastatic prostate cancer

**DOI:** 10.18632/oncotarget.27855

**Published:** 2021-01-05

**Authors:** Krastan B. Blagoev, Roumen Iordanov, Mengxi Zhou, Tito Fojo, Susan E. Bates

**Affiliations:** ^1^National Science Foundation, Alexandria, VA 22230, USA; ^2^Department of Biophysics, Johns Hopkins University, Baltimore, MD 21218, USA; ^3^Jackson Memorial Hospital, Department of Internal Medicine, Miami, FL 33136, USA; ^4^Department of Medicine, Division of Hematology/Oncology, Columbia University Medical Center, New York, NY 10032, USA; ^5^James J. Peters Veterans Affairs Medical Center, Bronx, NY 10032, USA

**Keywords:** drug resistance, metastatic prostate cancer, exponential growth, tumor kinetics, cancer stem cells

## Abstract

Most metastatic cancers develop drug resistance during treatment and continue to grow, driven by a subpopulation of cancer cells unresponsive to the therapy being administered. There is evidence that metastases are formed by phenotypically plastic cancer cells with stem-cell like properties. Currently the population structure and growth dynamics of the resulting metastatic tumors is unknown. Here, using scaling analysis of clinical data of tumor burden in patients with metastatic prostate cancer, we show that the drug resistant, metastasis-causing cells (MCC) are capable of producing drug resistant, exponentially growing tumors, responsible for tumor growth as a patient receives different treatments.

## INTRODUCTION

Tissue homeostasis is maintained by a small number of stem cells capable of symmetrical and asymmetrical cell division, with symmetrical cell division ensuring that the number of stem cells remains constant when a stem cell is lost and asymmetrical cell division maintaining the stem cell number constant when all stem cells are present. These stem cells produce more differentiated expanding progenitor cells which are replaced periodically (e.g., the colon is renewed every four days). Currently it is thought that cancers emerge from one or both of these two cell populations and cancer cells possessing stem cell markers and capable of proliferating when grafted in animals models [1–3] have been identified in leukemia and solid tumors, and have been called cancer stem cells (CSC) [4–6]. The principal cause of death in cancers is caused by the emergence of multidrug resistance in metastatic tumors. Currently an active area of research is aimed at characterizing the type of cell(s) from the primary tumor that is/are capable of causing metastasis and the type of cell(s) that is/are multidrug resistant [7]. The epithelial to mesenchymal transition (EMT) has been implicated in cancer cells and there is evidence that the metastatic-capable cells are somewhere on the spectrum between fully epithelial and fully mesenchymal cells while the metastasizing cells are phenotypically plastic, and capable of moving along that spectrum [7]. Recently it was reported [8] that the WNT/ß-Catenin pathway drives prostate cancer cells into symmetric cell divisions, suggesting that these cancer cells have lost the ability to divide asymmetrically [9]. Here we show that this is also the case in patients with metastatic prostate cancer.

According to the Center for Disease Control, prostate cancer is the second most common cancer in men after non-melanoma skin cancer with 13% of men diagnosed with prostate cancer during their lifetime and 2–3% dying from it. The prostate produces fluid protecting the sperm and both the fluid and the sperm are squeezed in the urethra by the muscle tissue in the prostate. The prostate is the size of a walnut organ with a primary structure consisting of ducts. The inner surface of the ducts is covered with epithelial luminal cells, which are surrounded by epithelial basal cells and scattered among them are neuroendocrine cells. The stroma around the ducts is dynamic phenotypically and consists of many different types of supporting cells including fibroblasts, immune, and smooth muscle cells. The luminal cells express an androgen receptor and secrete growth factors as well as the prostate-specific antigen (PSA). The basal and neuroendocrine cells do not seem to be androgen dependent. The hormones testosterone and androstenedione are the main androgens which are produced by the testes and in small amounts by the adrenal gland and control the function of the prostate. Most prostate cancers are believed to originate in the luminal layer, but an aggressive type of prostate can evolve to a neuroendocrine phenotype [10]. The tumor microenvironment is essential for cell migration and metastasis [11]. The primary treatment for metastatic prostate cancer is androgen deprivation therapy. However, most patients progress to a next stage called castration resistant prostate cancer and alternative treatments are currently administered at this stage. In this paper we analyzed patient data consisting of serial in time measurements of the PSA levels under different treatments.

## RESULTS

### Drug resistant tumors grow exponentially in the majority of patients

We began by analyzing the growth of tumors in patients with metastatic prostate cancer using scaling analysis. The levels of prostate-specific antigen (PSA), was used as the measure of tumor quantity in patients with D0 and castration-resistant prostate cancer (CRPC). To extract the behavior of a physical quantity across a population of patients it is sometimes possible to scale the data of individual patients in such a way that the data from individual patients can be averaged and plotted on the same graph. This powerful procedure is used in statistical physics and other sciences and when possible, it reveals behavior that is hard to extract from the individual patient data.

In the majority of metastatic solid tumors including prostate cancer, an effective treatment leads initially to a fall or decay in tumor mass followed by growth of the tumor mass after a nadir quantity is reached. We previously showed [12] that the decay part is well characterized by an exponential decay, which confirms that observation. This observation is mathematically represented as *M_s_e* ^–*dt*^ = *M_s_/e ^dt^,* where *M*_s_ is the mass of the sensitive cells that are exponentially decaying at rate *d* over time *t*. We hypothesize that past the nadir, the majority of drug-sensitive cells have been killed and the tumor now increases in size as the still viable, drug-resistant cancer cells continue to grow. This then makes the decay term, *M*_s_ *e*^-dt^ = *M*_s_ */e ^dt^* small compared to the growth term that is also exponential and is represented mathematically by *M_r_e ^g t^*, again where *M_r_* is the mass of the resistant cells that are exponentially growing at rate *g* over time *t*. Thus, past the nadir, the decay term can be neglected, leaving only the exponential growth part *M_r_e ^g t^*. Here M_s_ and M_r_ are positive constants and *d* and *g* are the decay and growth rates, respectively. Given this hypothesis in each patient we have the patient’s M_nadir_ and growth rate *g*.

**Figure 1:**
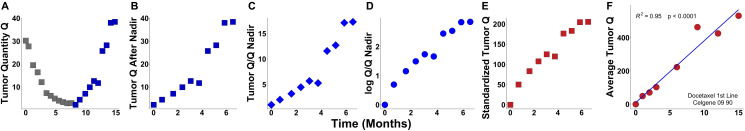
Example of data transformation during the scaling analysis. (**A**) Raw data that was obtained at pre-defined intervals of time; (**B**) Data after nadir; (**C**) Data after the nadir divided by the nadir value; (**D**) Log of step (C); (**E**) Standardized tumor quantity obtained by dividing step (D) by the slope or the rate constant of *g*rowth (*g*); (**F**) Average tumor quantity for all patients in the study. Note that steps (A–E) are plots for a single individual; while step (F) is the summary plot for the entire study. In all patients, data was obtained at pre-defined intervals of time, but these were not identical. To accommodate this variability when pooling data from hundreds of patients enrolled on a study [step (F)], a time window ± 15 days was employed. In this way, any data measured within ± 15 days is considered as one set of data.

To be able to plot all patients in a given trial on the same graph the data of each individual patient taken from the nadir was normalized to one by dividing each patient’s data by the value of the nadir of the patient. The resulting time series was transformed by taking the natural logarithm of the data of each patient. In Figure 1. We show the sequential transformation of a single patient’s data.

**Figure 2:**
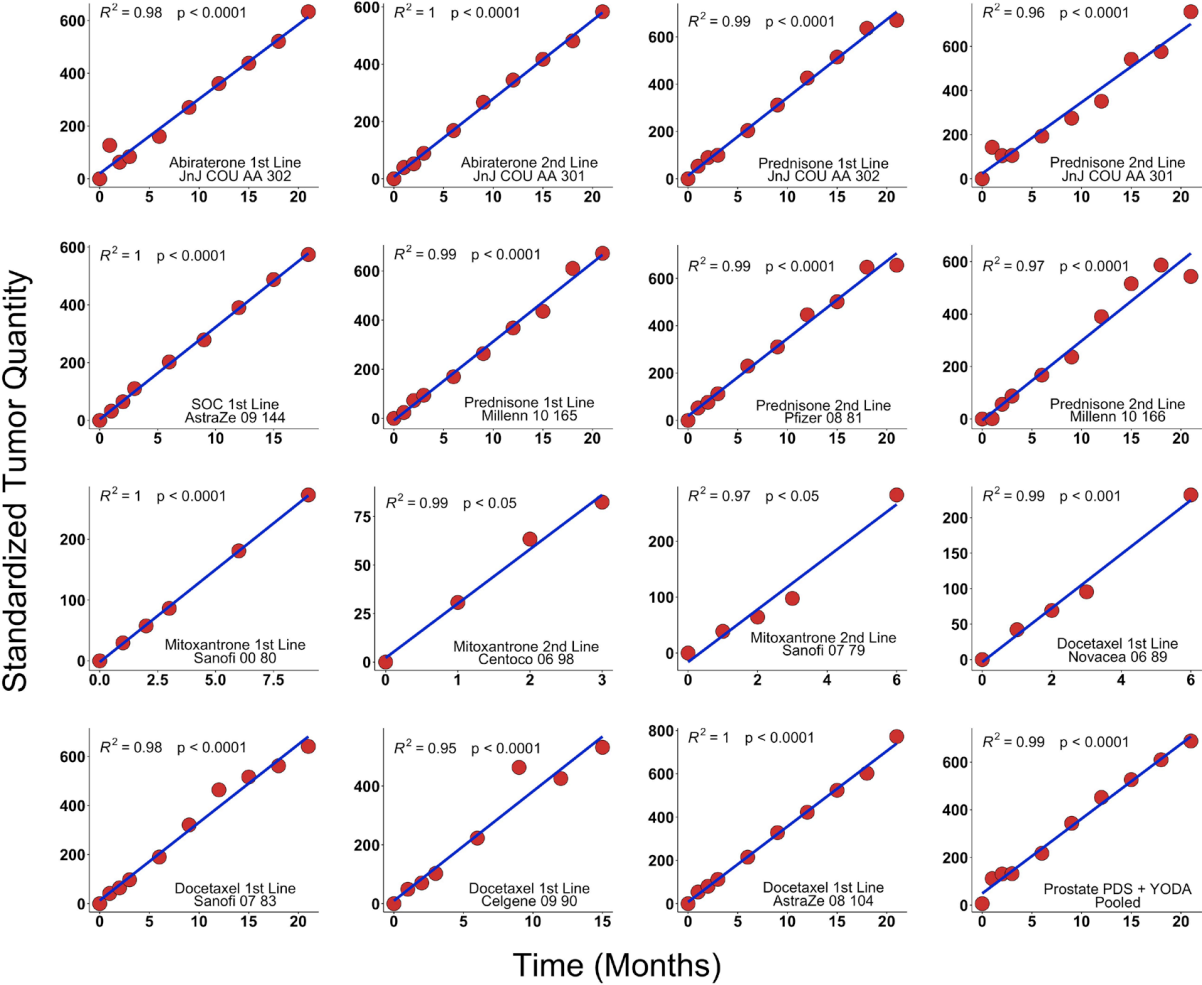
Results in clinical trials. PSA values from fifteen arms derived from prostate cancer clinical trials were analyzed using scaling analysis and averaging all patients in a treatment arm. The straight line indicates that tumor growth is exponential. These trials were summed in the lower right-hand corner (Methods). We analyzed de-identified, patient-level comparator (control) arm data from thirteen randomized metastatic castrate-resistant prostate cancer (mRPC) clinical trials including one phase IIB and ten phase III available in Project Data Sphere, LLC (PDS) and two randomized trials available from YODA (Yale University Open Data Access). Thirteen arms were the comparators and two treatment arms were abiraterone. All y-axes have the units of inverse rate, i.e., time.

If our hypothesis that tumors grow exponentially is correct a straight line would be a good fit of the resulting time series with a slope equal to the growth rate of each patient’s tumor. Therefore, the thus transformed data would consist of straight lines with different slopes. To be able to average all patients in any dataset we divide each transformed patient’s data by their growth rate (the slope of the straight line). After averaging the newly transformed data across all patients, we plot the scaled data on a graph. If our hypothesis is correct, averaging is possible since after normalizing the data to the tumor mass at the lowest point and scaling the logarithm of the data for each patient, by dividing it by the individual patient’s slope the resulting straight lines have the same slope and would lie on top of each other. In mathematical terms we divide each patient’s data by their M_nadir_, take the log and then divide by the patient’s *g*. These averages are presented in Figure 2. In these advanced cancers a straight line fits the logarithm of the data showing that our initial hypothesis of exponential growth is statistically plausible since growth other than exponential would have resulted in deviation from a straight line.

### Large number of symmetrically dividing cells is needed to explain the data

The simplest explanation for our results is symmetrical division of a cellular fraction with infinite or very large proliferative potential driving exponential tumor growth. Exponential and power law growth are only consistent with models that envision cells with an infinite or very large potential dividing either only symmetrically or asymmetrically as well as symmetrically. With > 90% of our data fit by an exponential growth model, our analysis unequivocally establishes for metastatic tumors in humans a model of cancer cells capable of dividing symmetrically leading to exponential or power law growth as the only biologically relevant models. Importantly, these non-linear functions fit the data over long periods of time, exceeding 1000 days in some patients, establishing that non-linear growth can occur over prolonged time intervals [13].

## DISCUSSION

The above analysis of the data from many prostate cancer patients who received different treatments showed that the metastatic drug resistant tumors grow exponentially—this exponential growth has to be driven by symmetrically dividing cells. These cells emerge as drug resistant and eventually cause death. In normal tissue the symmetry of cell division is maintained by extrinsic (environmental) and intrinsic factors. The environment where normal stem cells reside is the stem cell niche populated with supporting cells and having an organized tissue architecture. In contrast cancers lose the polarized structure of the cells present in normal tissue, grow as bulk cell masses, and break the basal membrane surrounding the prostate to metastasize to distant cites. This disorganization of tissue structure may be responsible for the loss of regulated asymmetric cell division [9] leading to tumor growth. Alternatively, mutations and epigenetic changes may lead to loss of function and symmetric cell division. Both mechanisms may be in play and further research is needed to characterize the architecture of the metastatic tumors and the operating cell division mechanisms. Currently the origin of these symmetrically dividing cells is unclear. One possibility is that one or more cancer stem cells begin to divide *symmetrically* overtaking the a*symmetrically* dividing cells. An alternative possibility is that one or more progenitor cells acquire infinite proliferation potential and drug resistance and overtake the tumor. Both of these transformations might be at play. Whether these extrinsic and intrinsic mechanisms operate alone or synergistically is impossible currently to say. Observation of the tumor at individual cell level as well as monitoring of molecular markers of individual cells will be necessary to resolve the drivers behind phenotypic cell decisions in normal and cancerous tissues. In the past decade significant progress in that direction was made using organotypic cultures [14] and intravital microscopy [15].

## MATERIALS AND METHODS

### Patient data

We collected patient level data from Project Data Sphere, LLC (PDS), an independent initiative of the CEO Roundtable on Cancer’s Life Sciences Consortium (http://www.projectdatasphere.org/); and from the Yale University Open Data Access Project (YODA) a project committed to supporting research focused on improving the health of patients and informing science and public health (http://www.yoda.yale.edu/). We analyzed two randomized trials available from YODA and de-identified, patient-level comparator (control) arm data from eleven randomized metastatic castrate-resistant prostate cancer (mCRPC) clinical trials (one phase IIB and ten phase III) available in PDS, a total of 15 arms. All of the data is available to anyone and requires only simple applications to gain access.

The YODA and PDS trials with control arms in italics are summarized in Table 1 and included: JnJ COU AA 302 [abiraterone + prednisone vs. prednisone] [16]; JnJ COU AA 301 [abiraterone + prednisone vs. prednisone] [17]; AstraZe 09 144 [ZD4054 (zibotentan) + SOC vs. SOC] [18]; Millen 10 165 [orteronel + prednisone vs. prednisone] [19]; Pfizer 08 81 [sunitinib + prednisone vs. prednisone] [20]; Millen 10 166 [orteronel + prednisone vs. prednisone] [21]; Sanofi 2000 80 [docetaxel + prednisone vs. mitoxantrone + prednisone] [22]; Centoco 2006 98 [siltuximab + mitoxantrone + prednisone vs. mitoxantrone + prednisone] [23]; Sanofi 2007 79 [TROPIC trial, cabazitaxel + prednisone vs. mitoxantrone + prednisone] [24]; Novacea 2006 89 [ASCENT trial, docetaxel + DN-101 vs. docetaxel + prednisone] [25]; Sanofi 2007 83 [VENICE trial, aflibercept + docetaxel + prednisone vs. docetaxel + prednisone] [26]; Celgene 2009 90 [MAINSAIL trial, lenalidomide + docetaxel + prednisone vs. docetaxel + prednisone] [27]; AstraZe 08 104; [docetaxel + zibotentan vs. docetaxel + placebo] [28]. All y-axes have the units of inverse rate.

**Table 1:** List of YODA and PDS trials whose data was analyzed

Data Source	Identifier	Line of therapy	Drug or placebo	Number of patients
**YODA**	COU_AA_302_PLB	First	Prednisone	542
	COU_AA_301_PLB	Second	Prednisone	398
	COU_AA_302_EXP	First	Abiraterone	546
	COU_AA_301_EXP	Second	Abiraterone	797
**PDS**	Prostat_AstraZe_2009_144	First	Placebo	266
	Prostat_Millenn_2010_165	First	Prednisone	779
	Prostat_Pfizer_2008_81	Second	Prednisone	285
	Prostat_Millenn_2010_166	Second	Prednisone	365
	Prostat_Sanofi_2000_80	First	Mitoxantrone	337
	Prostat_Centoco_2006_98	Second	Mitoxantrone	49
	Prostat_Sanofi_2007_79	Second	Mitoxantrone	371
	Prostat_Novacea_2006_89	First	Docetaxel	476
	Prostat_Sanofi_2007_83	First	Docetaxel	612
	Prostat_Celgene_2009_90	First	Docetaxel	526
	Prostat_AstraZe_2008_104	First	Docetaxel	470

### Estimating rates of tumor growth and regression

#### Model process

The rates of tumor growth and regression were estimated using an R package, designated tumgr, that uses a regression-growth model previously validated for other types of tumors and treatments [10, 26]. This model assumes that change from baseline in tumor quantity during therapy is the result of two independent processes occurring simultaneously: an exponential decay or regression of the tumor that is sensitive to the treatment and occurs at a constant rate, designated as *d*, and an exponential growth or regrowth of the tumor that is resistant or partially resistant to the treatment and likewise occurs at a constant rate and is designated as *g*. As each new quantity or measurement of tumor burden is estimated by summing the values for all individual lesions as they are accrued or in this analysis by measuring PSA levels, the value of g can be estimated in series. Previous data analysis has demonstrated that both the rate of tumor decay or regression (*d*) and the rates of growth (*g*) are stable or constant. Four possible models were defined:

#### gd model

There occurs concomitant regression of the sensitive fraction at rate *d* and growth of the resistant fraction at rate *g* and these are best fit by the *gd* model: f(t) = e ^−dt^ + e^gt^ − 1 where *f(t)* denotes the tumor quantity at time *t* in days, normalized to the tumor quantity at time 0; *d* is the rate of decay, and *g* is the rate of growth.

#### dx model

Tumors in which only decrease in tumor quantity at rate *d* occurs during treatment. These are best fit by the dx model since there is no growth (*g* = 0) and the *gd* equation then simplifies to:

f(t) = e^−dt^.

#### gx model

Tumors in which only increase in tumor quantity at rate *g* occurred during treatment are best fit by this model. Since there is no regression (*d* = 0) the *gd* equation then simplifies to:

f(t) = e^gt^

#### gdphi model

Similar to the *gd* model, in which during treatment there occurs concomitant regression of the sensitive fraction at rate *d* and growth of the resistant fraction at rate *g*, but in which the measurement data is very robust and one can better estimate the rates of growth and regression using an additional parameter, phi (Ø), which represents the fraction of tumor cells sensitive to therapy:

f(t) = (Ø)e^−dt^ + (1 − Ø)e^gt^

In this model, *d* denotes the rate of decay of the fraction of tumor sensitive to the therapy (Ø), and *g* represents the rate of growth of the therapy-resistant tumor fraction (1 − Ø).

#### Model analysis

Excluded from the analysis were patients without tumor measurements; or with only two data points. We also excluded those with only one unique measurement value repeated ≥ 3 times; and those with initial and final measurement values of 0. In some cases, the data could not be described by any of the four equations; while in others more than one equation could describe the data in a statistically meaningful way (*p* value for goodness of fit < 0.1). In these cases, the model with the lowest Akaike information criterion (AIC) was selected for each patient. The basis of AIC is information theory, and it provides a “relative estimate” of how much information is lost when a given model is used to represent data.

### Outcome

Our primary outcome of interest was the rates of tumor growth [*g*] and regression [*d*]. In the majority of metastatic solid tumors including prostate cancer, an effective treatment leads initially to a fall or *d*ecay in tumor mass that is sensitive to the treatment (*M*_s_) followed by *g*rowth of the tumor mass that is resistant to the therapy and is designated *M*_r_ after a nadir quantity is reached. We previously showed [12] that the decay part is well characterized by an exponential *d*ecay, which confirms that observation. This observation is mathematically represented as *M_s_* *e* ^–^^dt^ = *M*_*s*_ */* *e* *^dt^,* where *M_s_* is the mass of the sensitive cells that are exponentially *d*ecaying at rate *d* over time *t*. After initial decay, the PSA levels start to rise in most patients and the decay term becomes small compared to the *g*rowth (*g*) term and we focus on that term in the scaling analysis.

### Scaling

Scaling is a mathematical procedure in which a quantity at each time point is multiplied or divided by the same number. In this way the quantity is magnified or shrunk and the shape is changed. In our case the initial normalization of the data to 1 for each patient’s nadir and the division of the log of the normalized individual patient’s data by their growth rate are the two scaling procedures preformed in the analysis. Taking the log of the data is an example of functional transformation of the normalized data.

## Abbreviations

CRPC: castration-resistant prostate cancer
CSC: cancer stem cells
*d*: decay rate constant
EMT: epithelial to mesenchymal transition
*g*: growth rate constant
MCC: metastasis-causing cells
*M*_r_: *m*ass of cancer cells *r*esistant to a therapy
*M*_s_: *m*ass of cancer cells *s*ensitive to a therapy
PSA: prostate-specific antigen
*t*: time

## References

[1] Driessens G , Beck B , Caauwe A , Simons BD , Blanpain C . Defining the mode of tumour growth by clonal analysis. Nature. 2012; 488:527–30. 10.1038/nature11344. 22854777 PMC5553110

[2] Chen J , Li Y , Yu TS , McKay RM , Burns DK , Kernie SG , Parada LF . A restricted cell population propagates glioblastoma growth after chemotherapy. Nature. 2012; 488:522–6. 10.1038/nature11287. 22854781 PMC3427400

[3] Schepers AG , Snippert HJ , Stange DE , van den Born M , van Es JH , van de Wetering M , Clevers H . Lineage tracing reveals Lgr5+ stem cell activity in mouse intestinal adenomas. Science. 2012; 337:730–5. 10.1126/science.1224676. 22855427

[4] Lapidot T , Sirard C , Vormoor J , Murdoch B , Hoang T , Caceres-Cortes J , Minden M , Paterson B , Caligiuri MA , Dick JE . A cell initiating human acute myeloid leukaemia after transplantation into SCID mice. Nature. 1994; 367:645–8. 10.1038/367645a0. 7509044

[5] Reya T , Morrison SJ , Clarke MF , Weissman IL . Stem cells, cancer, and cancer stem cells. Nature. 2001; 414:105–11. 10.1038/35102167. 11689955

[6] Pattabiraman DR , Weinberg RA . Tackling the cancer stem cells - what challenges do they pose? Nat Rev Drug Discov. 2014; 13:497–512. 10.1038/nrd4253. 24981363 PMC4234172

[7] Dongre A , Weinberg RA . New insights into the mechanisms of epithelial-mesenchymal transition and implications for cancer. Nat Rev Mol Cell Biol. 2019; 20:69–84. 10.1038/s41580-018-0080-4. 30459476

[8] Zhang K , Guo Y , Wang X , Zhao H , Ji Z , Cheng C , Li L , Fang Y , Xu D , Zhu HH , Gao WQ . WNT/beta-Catenin Directs Self-Renewal Symmetric Cell Division of hTERT(high) Prostate Cancer Stem Cells. Cancer Res. 2017; 77:2534–47. 10.1158/0008-5472.can-16-1887. 28209613

[9] Venkei ZG , Yamashita YM . Emerging mechanisms of asymmetric stem cell division. J Cell Biol. 2018; 217:3785–95. 10.1083/jcb.201807037. 30232100 PMC6219723

[10] Hsu EC , Rice MA , Bermudez A , Marques FJG , Aslan M , Liu S , Ghoochani A , Zhang CA , Chen YS , Zlitni A , Kumar S , Nolley R , Habte F , et al. Trop2 is a driver of metastatic prostate cancer with neuroendocrine phenotype via PARP1. Proc Natl Acad Sci U S A. 2020; 117:2032–42. 10.1073/pnas.1905384117. 31932422 PMC6994991

[11] Ganguly SS , Li X , Miranti CK . The host microenvironment influences prostate cancer invasion, systemic spread, bone colonization, and osteoblastic metastasis. Front Oncol. 2014; 4:364. 10.3389/fonc.2014.00364. 25566502 PMC4266028

[12] Blagoev KB , Wilkerson J , Stein WD , Yang J , Bates SE , Fojo T . Therapies with diverse mechanisms of action kill cells by a similar exponential process in advanced cancers. Cancer Res. 2014; 74:4653–62. 10.1158/0008-5472.can-14-0420. 25183789 PMC8336537

[13] Burotto M , Wilkerson J , Stein W , Motzer R , Bates S , Fojo T . Continuing a cancer treatment despite tumor growth may be valuable: sunitinib in renal cell carcinoma as example. PLoS One. 2014; 9:e96316. 10.1371/journal.pone.0096316. 24796484 PMC4010463

[14] Gao D , Vela I , Sboner A , Iaquinta PJ , Karthaus WR , Gopalan A , Dowling C , Wanjala JN , Undvall EA , Arora VK , Wongvipat J , Kossai M , Ramazanoglu S , et al. Organoid cultures derived from patients with advanced prostate cancer. Cell. 2014; 159:176–87. 10.1016/j.cell.2014.08.016. 25201530 PMC4237931

[15] Mesa KR , Rompolas P , Zito G , Myung P , Sun TY , Brown S , Gonzalez DG , Blagoev KB , Haberman AM , Greco V . Niche-induced cell death and epithelial phagocytosis regulate hair follicle stem cell pool. Nature. 2015; 522:94–7. 10.1038/nature14306. 25849774 PMC4457634

[16] Ryan CJ , Smith MR , de Bono JS , Molina A , Logothetis CJ , de Souza P , Fizazi K , Mainwaring P , Piulats JM , Ng S , Carles J , Mulders PF , Basch E , et al, and COU-AA-302 Investigators. Abiraterone in metastatic prostate cancer without previous chemotherapy. N Engl J Med. 2013; 368:138–48. 10.1056/nejmoa1209096. 23228172 PMC3683570

[17] de Bono JS , Logothetis CJ , Molina A , Fizazi K , North S , Chu L , Chi KN , Jones RJ , Goodman OB Jr , Saad F , Staffurth JN , Mainwaring P , Harland S , et al, and COU-AA-301 Investigators. Abiraterone and increased survival in metastatic prostate cancer. N Engl J Med. 2011; 364:1995–2005. 10.1056/nejmoa1014618. 21612468 PMC3471149

[18] A Phase III Trial to Test the Efficacy of ZD4054 (Zibotentan), an Endothelin A Receptor Antagonist, Versus Placebo in Patients With Hormone Resistant Prostate Cancer (HRPC) and Bone Metastasis Who Are Pain Free and Mildly Symptomatic. AstraZe_2009_144. NCT00554229 https://clinicaltrials.gov/ct2/show/NCT00554229.

[19] Saad F , Fizazi K , Jinga V , Efstathiou E , Fong PC , Hart LL , Jones R , McDermott R , Wirth M , Suzuki K , MacLean DB , Wang L , Akaza H , et al, and ELM-PC 4 investigators. Orteronel plus prednisone in patients with chemotherapy-naive metastatic castration-resistant prostate cancer (ELM-PC 4): a double-blind, multicentre, phase 3, randomised, placebo-controlled trial. Lancet Oncol. 2015; 16:338–48. 10.1016/s1470-2045(15)70027-6. 25701170

[20] Michaelson MD , Oudard S , Ou YC , Sengelov L , Saad F , Houede N , Ostler P , Stenzl A , Daugaard G , Jones R , Laestadius F , Ullen A , Bahl A , et al. Randomized, placebo-controlled, phase III trial of sunitinib plus prednisone versus prednisone alone in progressive, metastatic, castration-resistant prostate cancer. J Clin Oncol. 2014; 32:76–82. 10.1200/jco.2012.48.5268. 24323035

[21] Fizazi K , Jones R , Oudard S , Efstathiou E , Saad F , de Wit R , De Bono J , Cruz FM , Fountzilas G , Ulys A , Carcano F , Agarwal N , Agus D , et al. Phase III, randomized, double-blind, multicenter trial comparing orteronel (TAK-700) plus prednisone with placebo plus prednisone in patients with metastatic castration-resistant prostate cancer that has progressed during or after docetaxel-based therapy: ELM-PC 5. J Clin Oncol. 2015; 33:723–31. 10.1200/jco.2014.56.5119. 25624429 PMC4879718

[22] Berthold DR , Pond GR , Soban F , de Wit R , Eisenberger M , Tannock IF . Docetaxel plus prednisone or mitoxantrone plus prednisone for advanced prostate cancer: updated survival in the TAX 327 study. J Clin Oncol. 2008; 26:242–5. 10.1200/jco.2007.12.4008. 18182665

[23] Fizazi K , De Bono JS , Flechon A , Heidenreich A , Voog E , Davis NB , Qi M , Bandekar R , Vermeulen JT , Cornfeld M , Hudes GR . Randomised phase II study of siltuximab (CNTO 328), an anti-IL-6 monoclonal antibody, in combination with mitoxantrone/prednisone versus mitoxantrone/prednisone alone in metastatic castration-resistant prostate cancer. Eur J Cancer. 2012; 48:85–93. 10.1016/j.ejca.2011.10.014. 22129890

[24] de Bono JS , Oudard S , Ozguroglu M , Hansen S , Machiels JP , Kocak I , Gravis G , Bodrogi I , Mackenzie MJ , Shen L , Roessner M , Gupta S , Sartor AO , and TROPIC Investigators. Prednisone plus cabazitaxel or mitoxantrone for metastatic castration-resistant prostate cancer progressing after docetaxel treatment: a randomised open-label trial. Lancet. 2010; 376:1147–54. 10.1016/s0140-6736(10)61389-x. 20888992

[25] Scher HI , Jia X , Chi K , de Wit R , Berry WR , Albers P , Henick B , Waterhouse D , Ruether DJ , Rosen PJ , Meluch AA , Nordquist LT , Venner PM , et al. Randomized, open-label phase III trial of docetaxel plus high-dose calcitriol versus docetaxel plus prednisone for patients with castration-resistant prostate cancer. J Clin Oncol. 2011; 29:2191–8. 10.1200/jco.2010.32.8815. 21483004

[26] Tannock IF , Fizazi K , Ivanov S , Karlsson CT , Flechon A , Skoneczna I , Orlandi F , Gravis G , Matveev V , Bavbek S , Gil T , Viana L , Arén O , et al, and VENICE Investigators. Aflibercept versus placebo in combination with docetaxel and prednisone for treatment of men with metastatic castration-resistant prostate cancer (VENICE): a phase 3, double-blind randomised trial. Lancet Oncol. 2013; 14:760–8. 10.1016/s1470-2045(13)70184-0. 23742877

[27] Petrylak DP , Vogelzang NJ , Budnik N , Wiechno PJ , Sternberg CN , Doner K , Bellmunt J , Burke JM , de Olza MO , Choudhury A , Gschwend JE , Kopyltsov E , Flechon A , et al. Docetaxel and prednisone with or without lenalidomide in chemotherapy-naive patients with metastatic castration-resistant prostate cancer (MAINSAIL): a randomised, double-blind, placebo-controlled phase 3 trial. Lancet Oncol. 2015; 16:417–25. 10.1016/s1470-2045(15)70025-2. 25743937

[28] Fizazi K , Higano CS , Nelson JB , Gleave M , Miller K , Morris T , Nathan FE , McIntosh S , Pemberton K , Moul JW . Phase III, randomized, placebo-controlled study of docetaxel in combination with zibotentan in patients with metastatic castration-resistant prostate cancer. J Clin Oncol. 2013; 31:1740–7. 10.1200/jco.2012.46.4149. 23569308

